# Post-COVID syndrome prevalence: a systematic review and meta-analysis

**DOI:** 10.1186/s12889-024-19264-5

**Published:** 2024-07-04

**Authors:** Ruhana Sk Abd Razak, Aniza Ismail, Aznida Firzah Abdul Aziz, Leny Suzana Suddin, Amirah Azzeri, Nur Insyirah Sha’ari

**Affiliations:** 1 Department of Public Health Medicine, Faculty of Medicine, Universiti Kebangsaan Malaysia, Bandar Tun Razak, Cheras, Kuala Lumpur 56000 Malaysia; 2 Faculty of Public Health, Universitas Sumatera Utara, Jalan Universitas No. 21 Kampus USU, Medan, North Sumatra 20155 Indonesia; 3 Department of Family Medicine, Faculty of Medicine, Universiti Kebangsaan Malaysia, Bandar Tun Razak, Cheras, Kuala Lumpur 56000 Malaysia; 4 Department of Public Health Medicine, Faculty of Medicine, Universiti Teknologi (UiTM) MARA, Sungai Buloh, Selangor Malaysia; 5 Department of Primary Care, Faculty of Medicine and Health Sciences, Universiti Sains Islam Malaysia (USIM), Persiaran Ilmu, Putra Nilai, Nilai, Negeri Sembilan 71800 Malaysia

**Keywords:** Post-COVID syndrome, COVID-19, Long COVID, Prevalence

## Abstract

**Background:**

Since the Coronavirus disease 2019 (COVID-19) pandemic began, the number of individuals recovering from COVID-19 infection have increased. Post-COVID Syndrome, or PCS, which is defined as signs and symptoms that develop during or after infection in line with COVID-19, continue beyond 12 weeks, and are not explained by an alternative diagnosis, has also gained attention. We systematically reviewed and determined the pooled prevalence estimate of PCS worldwide based on published literature.

**Methods:**

Relevant articles from the Web of Science, Scopus, PubMed, Cochrane Library, and Ovid MEDLINE databases were screened using a Preferred Reporting Items for Systematic Reviews and Meta-Analyses-guided systematic search process. The included studies were in English, published from January 2020 to April 2024, had overall PCS prevalence as one of the outcomes studied, involved a human population with confirmed COVID-19 diagnosis and undergone assessment at 12 weeks post-COVID infection or beyond. As the primary outcome measured, the pooled prevalence of PCS was estimated from a meta-analysis of the PCS prevalence data extracted from individual studies, which was conducted via the random-effects model. This study has been registered on PROSPERO (CRD42023435280).

**Results:**

Forty eight studies met the eligibility criteria and were included in this review. 16 were accepted for meta-analysis to estimate the pooled prevalence for PCS worldwide, which was 41.79% (95% confidence interval [CI] 39.70–43.88%, I^2^ = 51%, *p* = 0.03). Based on different assessment or follow-up timepoints after acute COVID-19 infection, PCS prevalence estimated at ≥ 3rd, ≥ 6th, and ≥ 12th months timepoints were each 45.06% (95% CI: 41.25–48.87%), 41.30% (95% CI: 34.37–48.24%), and 41.32% (95% CI: 39.27–43.37%), respectively. Sex-stratified PCS prevalence was estimated at 47.23% (95% CI: 44.03–50.42%) in male and 52.77% (95% CI: 49.58–55.97%) in female. Based on continental regions, pooled PCS prevalence was estimated at 46.28% (95% CI: 39.53%-53.03%) in Europe, 46.29% (95% CI: 35.82%-56.77%) in America, 49.79% (95% CI: 30.05%-69.54%) in Asia, and 42.41% (95% CI: 0.00%-90.06%) in Australia.

**Conclusion:**

The prevalence estimates in this meta-analysis could be used in further comprehensive studies on PCS, which might enable the development of better PCS management plans to reduce the effect of PCS on population health and the related economic burden.

**Supplementary Information:**

The online version contains supplementary material available at 10.1186/s12889-024-19264-5.

## Background

The novel severe acute respiratory syndrome coronavirus 2 (SARS-CoV-2) that first emerged in December 31st 2019 in Wuhan, China, causes the infectious coronavirus disease 2019 (COVID-19) [1, 2]. The World Health Organization (WHO) declared COVID-19 a Public Health Emergency of International Concern (PHEIC) on 30 January 2020, then a pandemic on 11 March 2020 [3, 4]. The COVID-19 pandemic has resulted in an increasing number of people recovering from SARS-CoV-2 acute infection [5]. COVID-19 patients might typically recover within a few weeks after symptom onset. However, some patients might experience health-related effects in the longer-term. Widely known as long COVID and post-COVID-19 condition, the conditions that occur post-COVID infection are also referred to with other terms, namely PCS, post-COVID-19 syndrome, long-haul COVID, post-acute COVID-19, long-term effects of COVID, or chronic COVID [6–12]. The WHO defined the post-COVID-19 condition as symptoms that occur at least 3 months after probable or confirmed SARS-CoV-2 infection that persist for at least 2 months and cannot be explained by an alternative diagnosis [13]. The symptoms might fluctuate, relapse, persist from the initial infection, or might also be new-onset after recovery from the acute illness [13]. In a COVID-19 rapid guideline, the National Institute for Health and Care Excellence (NICE), the Royal College of General Practitioners (RCGP), and the Scottish Intercollegiate Guidelines Network (SIGN) classified long COVID as “ongoing symptomatic COVID-19” and “post-COVID-19 syndrome”. Ongoing symptomatic COVID-19 is defined as signs and symptoms that persist 4–12 weeks after acute COVID-19, while post-COVID-19 syndrome is defined as signs and symptoms that develop during or after an infection in line with COVID-19 that continue for > 12 weeks and are not explained by an alternate diagnosis [14]. Given the increasing number of COVID-19 survivors, the above terms have gained widespread recognition in the scientific and medical communities [10, 11].

Post-recovery symptoms have become of increasing concern to more COVID-19 survivors [6]. Several studies have determined that COVID-19 exerts a wide range of long-term effects on virtually all body systems, including the respiratory, cardiovascular, neurological, gastrointestinal, psychiatric, and dermatological systems [6]. Cough and fatigue are among the multiorgan symptoms described following COVID-19 infection, as are shortness of breath, headache, palpitations, chest discomfort, joint pain, physical limits, depression, and insomnia [7]. A published review revealed that hepatic and gastrointestinal (*n* = 6), cardiovascular (*n* = 9), musculoskeletal and rheumatologic (*n* = 22), respiratory (*n* = 27), and neurologic and psychiatric (*n* = 41) issues were the most prevalent late complications which might occur post COVID-19 infection [15]. Certain risk factors such as older age and biological sex cannot be changed, thus management of other preventable and manageable risk factors like chronic comorbidities, may benefit the high-risk people from developing the persistent COVID-19 symptoms, even after few months post-acute COVID-19 infection. Epidemiological studies and related clinical trials addressing leading hypotheses may aid in the development of good management practices, including effective prevention and early intervention strategies to control the risk factors and manage the complications [16]. Regular disease surveillance and monitoring, implementation of related health promotion strategies, plus continuous efforts in researching for the best vaccines and treatment options may help in lowering the prevalence of PCS [17, 18].

An increasing number of published studies have focused on PCS. However, robust studies on this dynamic post-COVID condition are still required to identify the risk factors; explore the underlying aetiology; and plan strategies for preventative, rehabilitation, clinical, and public health management to enhance COVID-19 recovery and long-term outcomes [12]. Such studies should be conducted using the most recent data on PCS prevalence. Therefore, the present study systematically reviewed and determined the pooled prevalence of PCS worldwide based on current published literature.

## Methods

### Study design

Articles related to PCS and the prevalence data available worldwide were obtained using the Preferred Reporting Items for Systematic Reviews and Meta-Analyses (PRISMA) framework. The review protocol was registered with PROSPERO (CRD42023435280). All authors have a background in the related field and contributed collectively to meeting the study objective. The research question was developed, then a systematic search was conducted to identify and screen eligible studies based on the inclusion and exclusion criteria. Articles were identified from five primary databases. Relevant information and data were extracted from available full-text primary articles to answer the research question. The methodological quality of the included articles was assessed with the Joanna Briggs Institute (JBI) critical appraisal checklist. Subsequently, a meta-analysis was conducted to estimate the pooled prevalence of PCS worldwide.

### Outcomes and measures

The overall prevalence estimates of any persistent health conditions and symptoms at ≥ 12 weeks after the index date were set as the primary outcome. The 12-week timeframe was adopted to conform with the clinical definition of PCS, which is symptoms and signs that develop after or during infection consistent with COVID-19, not clarified by different diagnosis, and continue beyond 12 weeks.

### Inclusion and exclusion criteria

A set of inclusion and exclusion criteria was utilized as a basis for the identification and selection of relevant articles for this systematic review and meta-analysis study. The inclusion criteria were: 1) availability of full text; 2) article was written in English language; 3) article was published within 1 January 2020 to 27 April 2024; 4) study was related to prolonged post-COVID-19 conditions, and used human populations with COVID-19 diagnosis confirmed using PCR, antibody testing, or a clinical diagnosis; 5) study had an index date using the COVID-19 onset date, first test or diagnosis, hospitalisation date, or discharge date; and 6) study had adequate data on the estimates of overall PCS prevalence in a community, i.e. studies which not only focused on the prevalence of a specific PCS symptom as their only outcome. This was to ensure that the primary outcome in this meta-analysis, which is the pooled overall prevalence of PCS is derived only from studies with identical outcomes, besides limiting the probabilities of any bias resulting from including studies which only published symptom-specific PCS prevalence data estimates. Another inclusion criteria used was 7) assessment date, or follow-up or clinical check-up date at least 12 weeks after the index date. Meanwhile, the exclusion criteria were non-accessible articles and publications with content unrelated to the research question. Non-primary publications such as book chapters or letters to editor, and case reports were also excluded.

### Search strategy

The search terms used in the article identification stage were derived from medical subject heading (MeSH) terms and synonyms related to the review topic. Then, two authors (RR and NIS) conducted a systematic search of the abovementioned databases using the search strings developed from combining the identified search terms and Boolean operators. The search string used was (("PCS" OR "post COVID syndrome" OR "post COVID-19 syndrome" OR "post COVID condition*" OR "post COVID-19 condition*" OR "post COVID" OR "post-COVID" OR "post COVID-19" OR "post-COVID-19" OR "post COVID sequela" OR "post-COVID sequela" OR "post COVID sequelae" OR "post-COVID sequelae" OR "long COVID" OR "long-COVID" OR "long haul*" OR "long-haul*" OR "long COVID-19" OR "long-COVID-19" OR "covid syndrome" OR "covid-19 syndrome" OR "post-acute COVID-19 syndrome" OR "post acute COVID-19" OR "post acute COVID" OR "chronic COVID" OR "chronic COVID-19" OR "persistent COVID" OR "persistent post-COVID" OR "persistent post COVID" OR "prolonged COVID" OR "prolonged post-COVID" OR "prolonged post COVID") AND ("prevalence*")). Available filters based on the inclusion and exclusion criteria were applied during the database search.

### Data sources

Relevant articles searched and identified from five databases (Web of Science [WOS], Scopus, PubMed, Cochrane Library, Ovid MEDLINE) on 29 April 2024, were downloaded by author RR and collected in Mendeley Desktop version 1.19.8. Subsequently, duplicates were identified and removed by author NIS, and the shortlisted articles were transferred to Microsoft Excel for further screening.

### Study selection

Relevant studies were selected via a screening process conducted by two authors, who independently screened the article titles and abstracts, then retrieved the full text of shortlisted articles. Efforts to include all available studies were made and included accessing publications via institutional accounts. Subsequently, two authors (RR and NIS) examined the full texts of potential eligible papers separately, followed by discussions and re-evaluation among them to resolve any contradictory decisions. A third author (AI) was also employed in this process, when there are uncertainties in the decision-making process.

### Data extraction

Two authors (RR and NIS) then extracted and tabulated the relevant data elements (article title, authors, publication year, study design, country, study population, study setting, sample size and number of cases identified, duration from index to assessment date, PCS prevalence estimates).

### Methodological quality assessment

The methodological quality of the studies was evaluated with the Joanna Briggs Institute (JBI) Critical Appraisal Checklist for Studies Reporting Prevalence Data to ascertain how well the article addressed the possibility of bias. All articles screened and selected for inclusion in this systematic review were appraised by two critical appraisers (RR and NIS). The JBI checklist contains 9 items which comprised of 1 question each; (Item 1: Was the sample frame appropriate to address the target population?), (Item 2: Were study participants sampled in an appropriate way?), (Item 3: Was the sample size adequate?), (Item 4: Were the study subjects and the setting described in detail?), (Item 5: Was the data analysis conducted with sufficient coverage of the identified sample?), (Item 6: Were valid methods used for the identification of the condition?), (Item 7: Was the condition measured in a standard, reliable way for all participants?), (Item 8: Was there appropriate statistical analysis?), (Item 9: Was the response rate adequate, and if not, was the low response rate managed appropriately?). Each item is coded as “yes/no/unclear/not applicable”. Each of these items is assessed by scoring (yes = 1), (no = 0), and (unclear or not applicable = 0). The total score of each included study was presented as percentages, which then categorized into 3 levels of risk of bias: (20–50% items scored yes = high risk of bias), (50–80% items scored yes = moderate risk of bias), and (80–100% items scored yes = low risk of bias). Based on the assessment result, both appraisers discussed and finalised the decision on the overall appraisal, i.e., whether to include the assessed study in the review.

### Statistical analysis

The meta-analysis was conducted using the metaprop function in the R 4.3.1 meta package. Due to the heterogeneity of the included studies as resulted from differences in studied populations’ factors, varied geographical regions and PCS assessment timepoints, a random-effects model was considered as the better choice for assigning weights to each study in the meta-analysis. The pooled prevalence and effect sizes for each study were included in a forest plot, where the size of each study was proportional to its weight. Statistical heterogeneity was measured with I^2^ statistics versus p-values, where a p-value of 0.05 and an I^2^ of ≥ 50% indicated high heterogeneity. Visual inspection of the generated funnel plot’s symmetricity was conducted to determine any influence of publication bias on the findings. Egger’s test and Begg rank correlation test were also conducted for further identification of the presence of any asymmetricities.

## Results

### Study selection

**Fig. 1:**
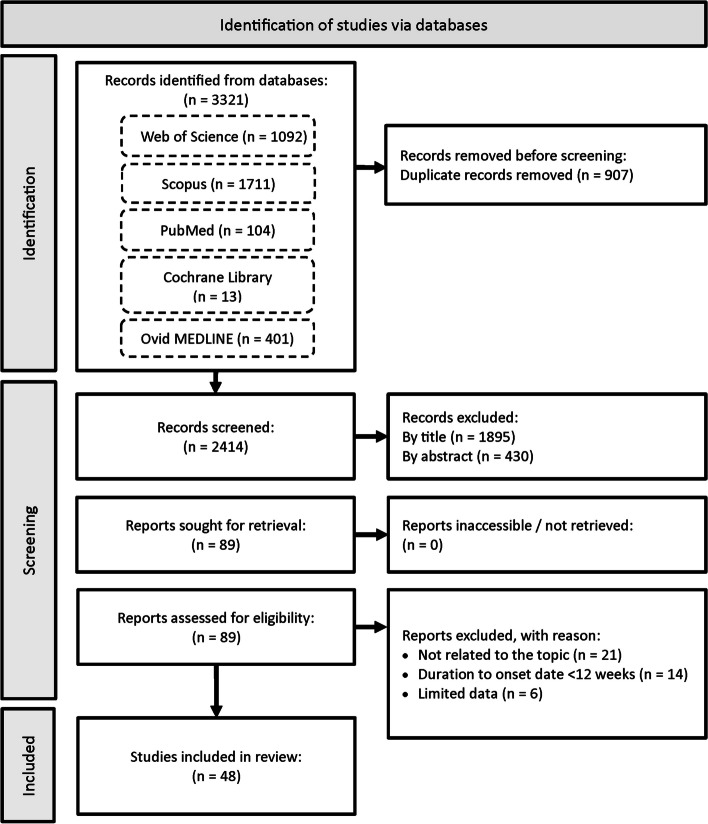
Flowchart showing the study selection process and number of results

Overall, a total of 3321 records were identified from the main literature search conducted in end of April 2024, of which 907 duplicate articles were removed. Screening of the article titles and abstracts resulted in 2325 articles unrelated to the research question being excluded. All remaining articles were retrieved to determine their accessibility, of which 89 successfully retrieved full-text articles were reviewed and assessed for eligibility. Articles with contents not relevant to this study were excluded. Studies with sample populations with mean or median prolonged signs or symptoms, or health care utilisation, or follow-up time < 12 weeks from acute COVID-19 symptom onset were excluded to ensure that the samples with persistent COVID-19 symptoms in the finalised studies met the definition of PCS. A total of 41 articles were excluded, as these studies and their contents did not align with the review topic or the other inclusion and exclusion criteria. Finally, 48 articles were included in this review. The PRISMA flow diagram in Fig. 1 depicts the literature selection process and search criteria, and the number of articles involved for each process.

### Study characteristics and PCS prevalence

Table 1 presents the study characteristics of the 48 included studies, including the overall PCS prevalence data from each study. Among those studies, 21 were from European countries, 14 studies were from American region, 10 were from Asia, two were from Australia, and one study from African continent. Forty one included studies were cohort studies, 5 were cross-sectional and 2 were case–control studies. The studies involved sample sizes of 106–124313 individuals diagnosed with COVID-19 at least 12 weeks prior to the assessment date. The index date-to-assessment date duration ranged from 12 weeks to 25.5 months. Among the included studies, 10 studies focused mainly on the previously hospitalized COVID-19 patients and 1 study researched on PCS among the non-hospitalized COVID-19 patients. Majority of the included studies studied both previously hospitalized and non-hospitalized COVID-19 patients, as shown in 35 studies in Table 1. Most of the examined populations in the 48 included studies were adult-aged, while the percentage of female participants varied from 26.5% to 77.5%.

**Table 1:** Summary of characteristics of the 48 included studies

**Authors**	**Year**	**Country**	**Continental Region**	**Study Design**^a^	**Study Population**	**Study Population Sex: Female (%)**	**Study Population Age**^b^	**Sample size**	**Post-COVID-19 Assessment Timepoints**^c^	**Prevalence (%)**	**Risk of Bias**^d^
Bellan M et al. [19]	2021	Italy	Southern Europe	C	Hospitalized COVID-19 patients	39.0	62	200	366 days	39.50	
Bliddal S et al. [20]	2021	Denmark	Northern Europe	C	Non-hospitalized COVID-19 patients	70.0	44.9	129	12 weeks	40.00	
Peghin M et al. [21]	2021	Italy	Southern Europe	C	Mixed	53.4	53 (15.8)	599	187 days	40.20	
Zayet S et al. [22]	2021	France	Western Europe	C	Mixed	63.0	49.6 (18.7)	354	289 days	35.90	
Fjelltveit EB et al. [23]	2022	Norway	Northern Europe	CC	Mixed	53.0	44	233	12 months	46.00	
Fumagalli C et al. [24]	2022	Italy	Southern Europe	C	Hospitalized COVID-19 patients	40.2	62 (15)	254	12 months	40.50	
Helmsdal G et al. [25]	2022	Denmark	Northern Europe	C	Mixed	54.7	41	170	22.6 months	38.00	
Kingery JR et al. [26]	2022	United States of America	Northern America	C	Mixed	44.5	59.2 (16.2)	530	332 days	44.20	
Knight DR et al. [27]	2022	United States of America	Northern America	C	Mixed	60.2	54	437	3–6 months	34.90	
Nehme M et al. [28]	2022	Switzerland	Western Europe	C	Non-hospitalized COVID-19 patients	64.8	44.2 (14.5)	287	12 months	33.40	
Petersen MS et al. [29]	2022	Denmark	Northern Europe	C	Mixed	54.7	35.4	170	168 days	39.00	
Rivera M et al. [30]	2022	Spain	Southern Europe	C	Hospitalized COVID-19 patients	42.6	61.2 (14.3)	453	12 months	36.10	
Tisler A et al. [31]	2022	Estonia	Northern Europe	C	Mixed	54.3	65.4 (16.7)	3949	294 days	40.30	
Titze R et al. [32]	2022	Brazil	Latin America and the Caribbean	C	Mixed	61.0	41.2 (19–81)	236	5–8 months	41.50	
Wu Q et al. [33]	2022	United States of America	Northern America	C	Mixed	57.3	46.0 (15.8)	308	12 weeks	23.00	
Babicki M et al. [34]	2023	Republic of Poland	Europe	C	Mixed	63.1	52 (43–63)	643	3 & 12 months	65.70	
Boglione L et al. [35]	2023	Italy	Europe	C	Hospitalized COVID-19 patients	26.5	66.5	306	25.5 months	43.80	
Daniel CL et al. [36]	2023	United States of America	America	C	Mixed	65.0	41.1 (25.3–54.6)	516	6 & 12 months	20.00	
Fatima S et al. [37]	2023	Pakistan	Asia	C	Hospitalized COVID-19 patients	38.3	56.9 (14.3)	459	12 months	29.90	
Feter N et al. [38]	2023	Brazil	America	C	Mixed	74.4	38.3 (11.9)	1001	3 months	77.42	
Gaspar P et al. [39]	2023	Portugal	Europe	C	Hospitalized COVID-19 patients	43.4	61	152	3,6,9 months	66.50	
Hastie C.E. et al. [40]	2023	Scotland, UK	Europe	C	Mixed	58.8	≥ 16	64,733	6 months	64.50	
Hua M.J. et al. [41]	2023	United States of America	America	C	Hospitalized COVID-19 patients	36.0	53.8 (14.1)	145	255 days	34.00	
Jayasekera M.M.P.T. et al. [42]	2023	Sri Lanka	Asia	C	Hospitalized COVID-19 patients	45.8	57.2 (16.3)	153	3,6,12 months	60.10	
Jogdand M.S. et al. [43]	2023	India	Asia	CS	Mixed	41.0	< 18 -59	200	3 months	17.50	
Khanafer N et al. [44]	2023	France	Europe	C	Hospitalized COVID-19 patients	40.2	63.4 (25–93)	189	8.8 months	57.70	
Kim Y et al. [45]	2023	South Korea	Asia	C	Mixed	68.2	38 (24,50.5)	132	819 days	71.20	
Krishnadath I et al. [46]	2023	Republic of Suriname	America	C	Mixed	62.3	49 (15)	106	3–4 months	39.60	
Lapa J et al. [47]	2023	Brazil	America	C	Hospitalized COVID-19 patients	48.4	57	400	3, 6 months	80.70	
Martínez-Ayala M.C. et al. [48]	2023	Colombia	America	C	Mixed	55.02	53	1723	6 months	47.07	
Montoy J.C.C. et al. [49]	2023	United States of America	America	C	Mixed	65.2	≥ 18	1017	3,6,9,12 months	48.20	
Peghin M et al. [50]	2023	Italy	Europe	C	Mixed	53.5	54.7	230	2.3 years	36.10	
Rodríguez Onieva A et al. [51]	2023	Spain	Europe	C	Mixed	55.8	45.8%: (44.93–46.66)	1542	90 days	12.39	
Silva KM et al. [52]	2023	Brazil	America	C	Mixed	50.7	39.7 (11.7)	1371	11.9 months	63.90	
Talhari C et al. [53]	2023	Brazil	Asia	CS	Mixed	69.9	66.9%: (31–60)	6958	12 weeks	83.20	
Tran TK et al. [54]	2023	Vietnam	Asia	C	Mixed	52.0	91.2%: (18–60)	125	6 & 9 months	12.00	
van der Maaden T et al. [55]	2023	Netherlands	Europe	CC	Mixed	63.7	49.0 (37–61)	9116	3 months	48.50	
Wahlgren C et al. [56]	2023	Sweden	Europe	C	Hospitalized COVID-19 patients	40.0	61 (13)	165	719 days	84.00	
Wong MCS et al. [57]	2023	China	Asia	CS	Mixed	60.0	80.5%: (25–44)	2712	3 months	90.40	
Bello-Chavolla OY et al. [58]	2024	Mexico	America	C	Mixed	55.0	43 (31,55)	5,211	6 months	21.21	
Gwaikolo C et al. [59]	2024	Liberia	Africa	C	Mixed	43.6	38 (30–49)	505	3, 6 months	50.20	
Jangnin R et al. [60]	2024	Thailand	Asia	C	Mixed	55.6	31.8 (13.6)	390	3–12 months	77.70	
Keng Tok P.S. et al. [61]	2024	Malaysia	Asia	C	Mixed	60.6	38.2 (11.9)	44,386	1,3,6 months	3.40	
Nguyen K.H. et al. [62]	2024	United States of America	America	CS	Mixed	53.5	60.7%: (18–49)	124,313	3 months	21.80	
Patro M et al. [63]	2024	India	Asia	C	Mixed	42.5	50.72 (13.7)	200	3,6,12 months	51.00	
Salmon D et al. [64]	2024	France	Europe	C	Mixed	77.5	71.9%: (≥ 40)	231	12 months	62.70	
Tan S et al. [65]	2024	Australia	Australia	C	Mixed	71.9	58 (19.4)	196	1,3,6,12 months	66.80	
Woldegiorgis M et al. [66]	2024	Australia	Australia	CS	Mixed	52.0	21%: (30–39)	11,697	90 days	18.20	

^a^For study designs, C: Cohort; CC: Case–Control, CS: Cross-Sectional study design

^b^Study age formats as published in the individual study, i.e.: mean/median/mean (SD)/mean (IQR)/ age group with the highest percentage / range of age group

^c^Post-COVID-19 Assessment Timepoints refers to the duration from index date to assessment date, which was mainly analyzed in the individual study and closest to the PCS term definition used in this study. Index date refers to the date of onset, first test or diagnosis, date of hospitalization, or discharge date. Assessment date also refers to the time of follow-up, or data collection

^d^Risk of Bias (based on Joanna Briggs Institute (JBI) Critical Appraisal Checklist):  (Red) = High risk of bias,  (Yellow) = Moderate risk of bias,  (Green) = Low risk of bias. [Further details described under the Methodological Quality Assessment section]

### Methodological quality assessment

Joanna Briggs Institute (JBI) Critical Appraisal Checklist for Studies Reporting Prevalence Data was used to assess the methodological quality of the included studies. The assessment results reflect the methodological quality and risk of bias levels of the individual studies, which were categorized into low (80%-100% scores), moderate (50%–80% scores), and high (20%–50% scores) risk of bias levels. The assessment result aids in finalizing the decision on the overall review of the individual studies, i.e., whether to include the assessed study in the review. Based on the checklist, majority of the 48 included articles in this review were of high methodological quality, with low risk of bias. The risk of bias levels for each study were listed under the last column titled ‘Risk of Bias’ in Table 1 (Summary of characteristics of the 48 included studies table). All 48 assessed studies were accepted to be included in this review.

### Pooled prevalence estimate of post-COVID syndrome

**Fig. 2:**
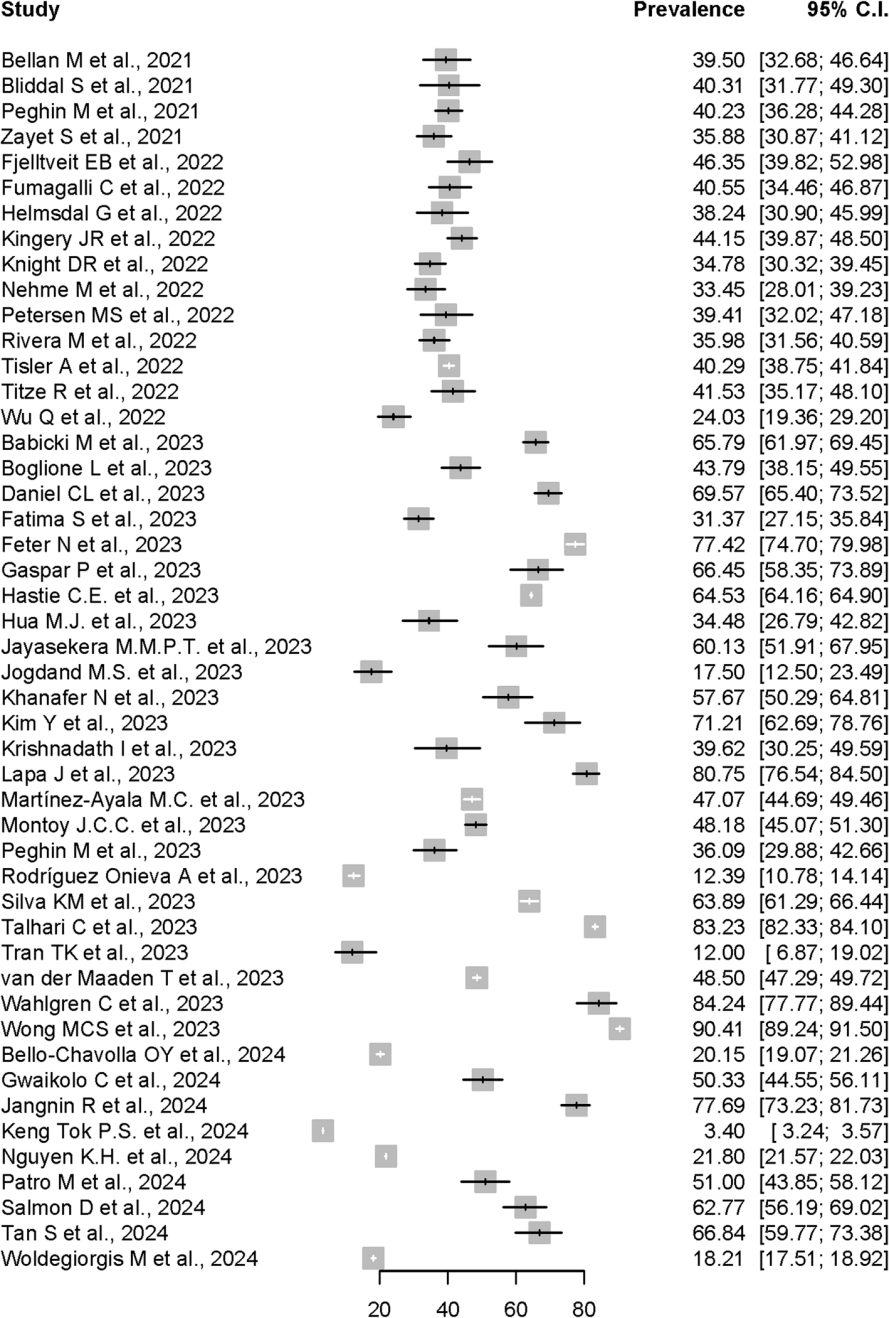
Forest plot presenting the Post-COVID Syndrome (PCS) prevalence data from all 48 studies

As shown by the forest plot in Fig. 2, the prevalence estimates of PCS reported in the 48 included individual studies ranged from 3.4% to 90.41%. Due to the significant high heterogeneity (I^2^ = 100%, *p* = 0) and presence of funnel plot asymmetry indicated by Egger’ test observed if meta-analysis was to be conducted using the prevalence data from all 48 included studies, only 16 studies were accepted for meta-analysis of the overall PCS prevalence, after excluding potential influential outliers based on the influence analyses done, including leave-one out analyses, risk of bias assessment for studies, and influential outliers.

**Fig. 3:**
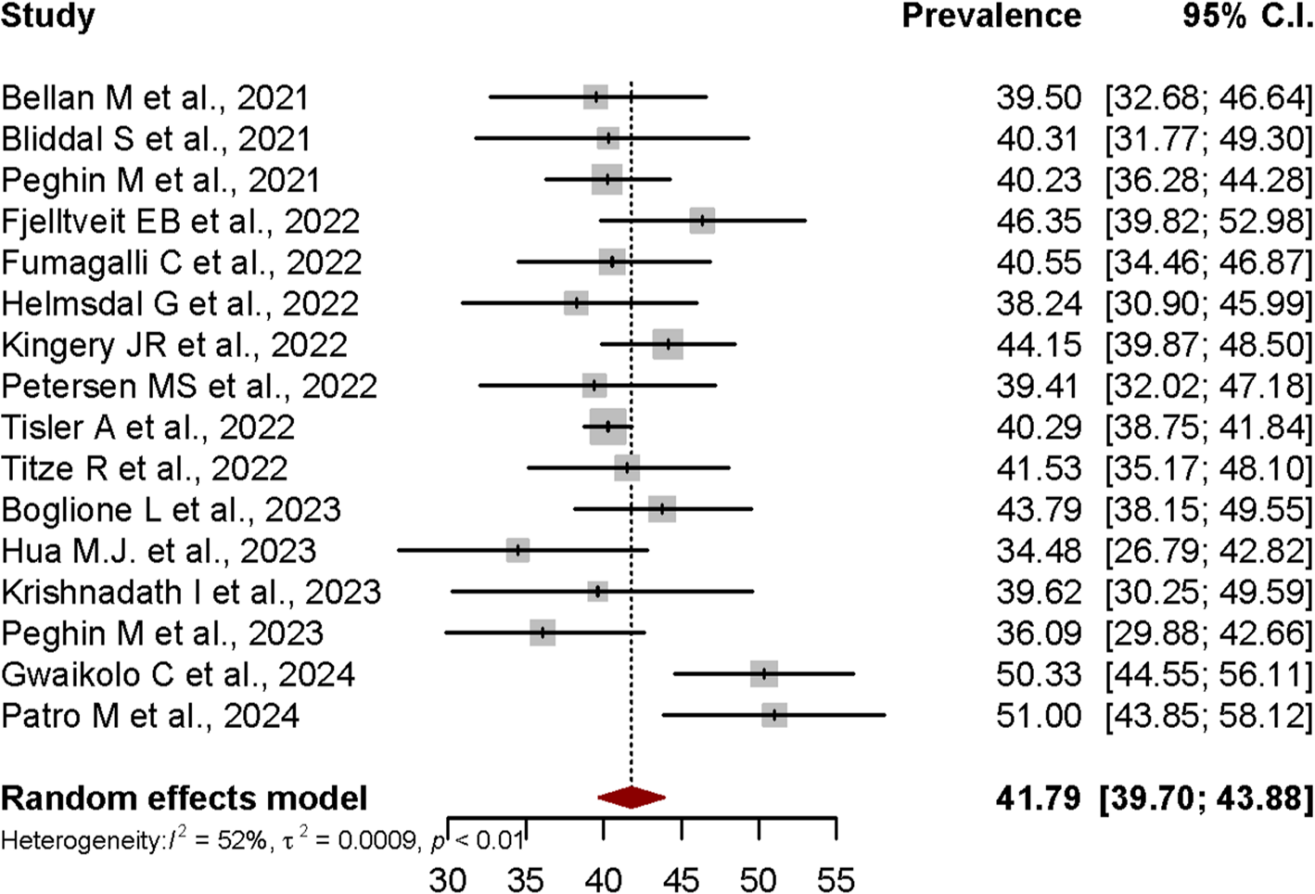
Forest plot presenting the Post-COVID Syndrome (PCS) pooled prevalence

**Fig. 4:**
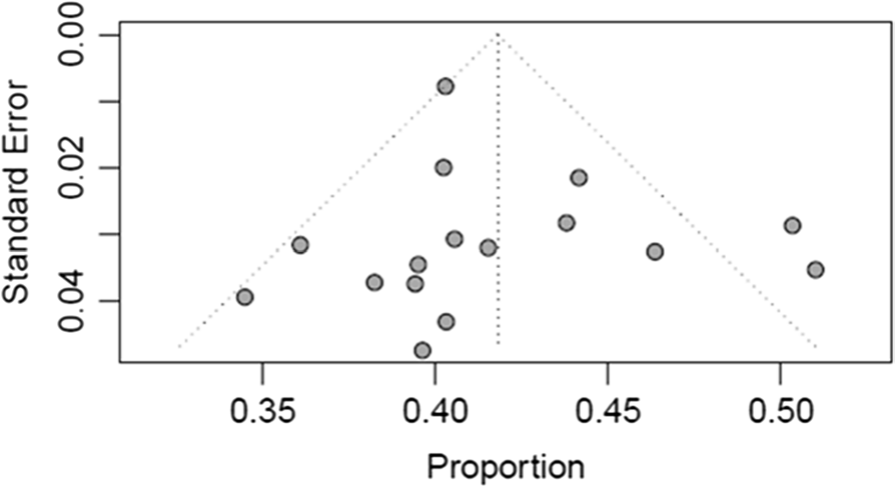
Funnel plot for the publication bias assessment of the 16 studies

In the meta-analysis conducted using the 16 allowed studies, the pooled prevalence of PCS estimated by random-effects model using data from the 16 studies was 41.79% (95% CI: 39.70%-43.88%). The forest plot shown in Figure 3 depicts the results derived from the random effects model, while Fig. 4 shows the funnel plot for the publication bias assessment of the 16 studies.

### Assessment of heterogeneity

Generally, heterogeneity is to be expected in a meta-analysis [67]. I^2^ was used to measure heterogeneity, with limits of ≥ 25%, ≥ 50%, and ≥ 75% each denoting low, moderate, and high heterogeneity. The meta-analysis conducted using random-effects model to calculate the pooled-prevalence of PCS in this study revealed significant mild to moderate heterogeneity across the included studies (I^2^ = 52%, *p* < 0.01). The variance in the underlying distribution of true effect sizes, or the between-study heterogeneity, was estimated at τ^2^ = 0.0009. In meta-analyses, heterogeneity is frequently unavoidable due to variations in study quality, methodology, sample size, and participant inclusion criteria [49, 68].

## Assessment of publication bias

Publication bias might occur when journals and authors only publish articles with the outcome of interest and can be detected by visual inspection of funnel plots. As shown in Fig. 4, publication bias was visually implied from the asymmetrical funnel plot. However, further analysis using Egger’s test did not indicate the presence of funnel plot asymmetry, although it was not statistically significant (*p* = 0.4661). Begg rank correlation test results was also not significant with p-value of 0.7871. These formal tests findings suggested that the results were not influenced by publication bias. Nevertheless, any visual asymmetry in the funnel plot might also be caused by true heterogeneity other than publication bias [69].

### PCS prevalence at different Post-COVID assessment timepoints

**Fig. 5:**
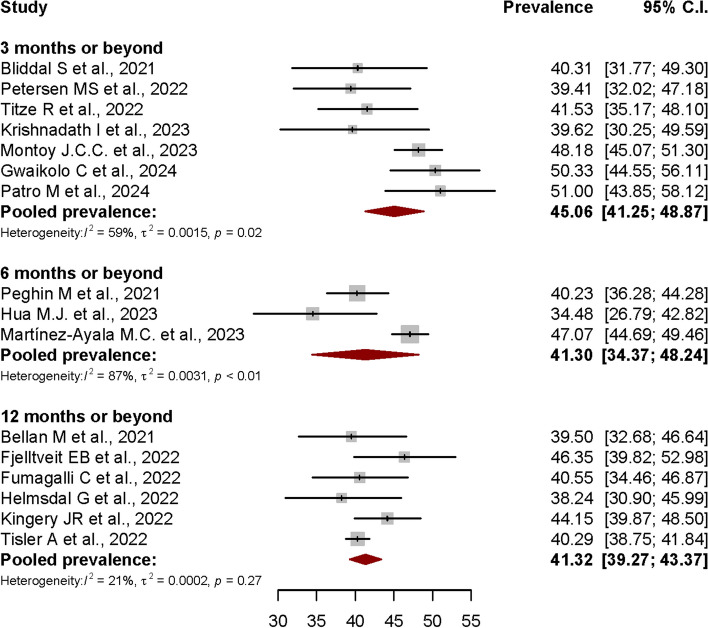
Forest plot showing the Post-COVID Syndrome prevalence at different assessment timepoints

To assess if the pooled prevalence of PCS was increasing over time after the acute COVID-19 infection, we stratified the included studies based on different assessment or follow-up timepoints. A subgroup analysis was performed to get the PCS pooled prevalence at ≥ 3rd, ≥ 6th, and ≥ 12th months post-COVID-19 infection. As shown in Fig. 5, the estimated Post-COVID Syndrome pooled prevalences at ≥ 3rd, ≥ 6th, and ≥ 12th months timepoints were each 45.06% (95% CI: 41.25%-48.87%, I^2^ = 59%, *p* = 0.02), 41.30% (95% CI: 34.37%-48.24%, I^2^ = 87%, *p* < 0.01), and 41.32% (95% CI: 39.27%-43.37%, I^2^ = 21%, *p* < 0.27), respectively.

### Post-COVID syndrome prevalence in male and female

**Fig. 6:**
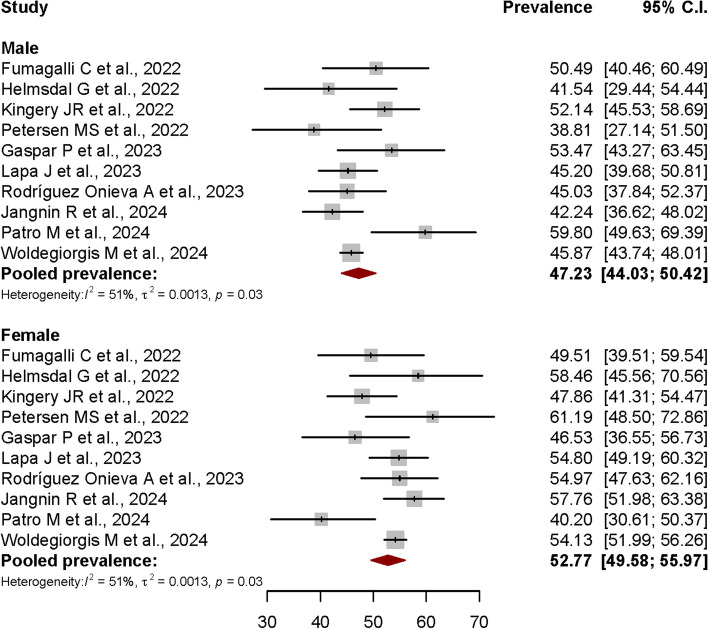
Forest plot showing the Post-COVID Syndrome prevalence in both male and female sex

Further subgroup analysis was conducted to examine the PCS prevalences among male and female. For this purpose, data from 10 articles out of all 48 included articles were allowed for the subgroup analysis, after the exclusion of influential outliers to estimate the pooled prevalences with less amount of heterogeneity. As shown in Fig. 6, the estimated Post-COVID Syndrome prevalence were 47.23% in male (95% CI: 44.03%–50.42%), and 52.77% in female (95% CI: 49.58%-55.97%). The studies had significant moderate heterogeneity with I^2^ = 51%, *p* = 0.03.

### Post-COVID syndrome prevalence in different continental regions

Another subgroup analysis based on stratification of PCS prevalence by continental regions was also performed. For this purpose, data from all 48 articles were included in the analysis.

**Fig. 7:**
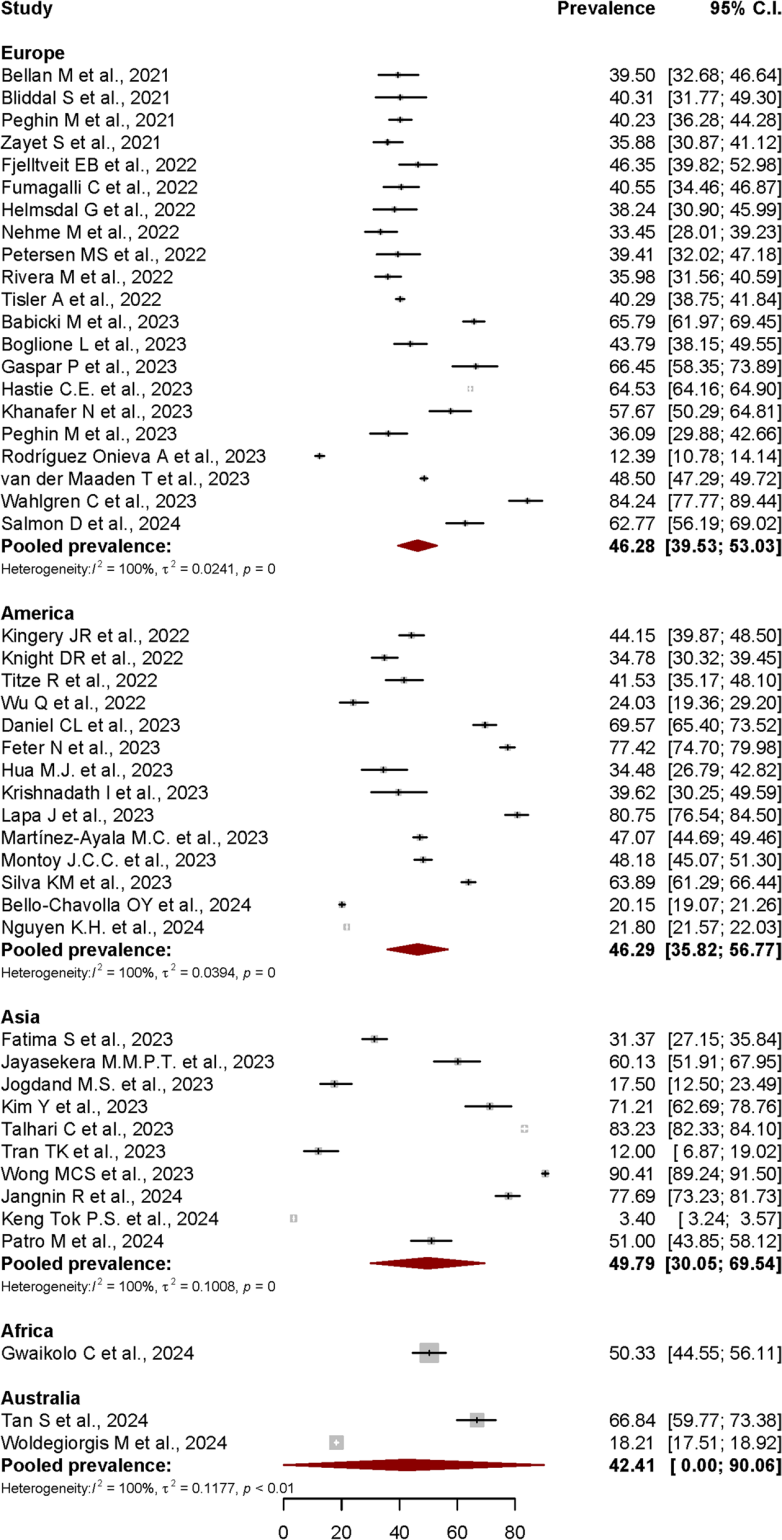
Forest plot showing the Post-COVID Syndrome prevalence in different continental regions

The estimated Post-COVID Syndrome prevalences according to the continental regions were shown in Fig. 7. The pooled prevalence was 46.28% (95% CI: 39.53%-53.03%) in Europe, 46.29% (95% CI: 35.82%-56.77%) in America, 49.79% (95% CI: 30.05%-69.54%) in Asia, and 42.41% (95% CI: 0.00%-90.06%) in Australia. Only one study from African continent was included in this review, with PCS prevalence reported at 50.33% (95% CI: 44.55%-56.11%). Most of the subgroups showed a significant heterogeneity level with I^2^ = 100%, *p* < 0.01.

## Discussion

### Post-COVID syndrome (PCS)

In this systematic review and meta-analysis, the term described by NICE; which defined PCS as signs and symptoms that develop during or after an infection in line with COVID-19 that continue for > 12 weeks and are not explained by an alternate diagnosis, was used as a basis to identify the overall PCS prevalence data [14] from published studies worldwide. The cut-off point of 12 weeks was strictly used to extract and analyse the relevant data during the systematic review process.

### Overall prevalence estimates of PCS worldwide

In this review, a total of 2414 published articles were screened from 3321 articles identified from 5 databases using a PRISMA-guided systematic search. The meta-analysis of 48 included studies that individually reported PCS prevalence data determined that the estimated pooled prevalence of PCS worldwide was 41.79% (95% CI: 39.70%-43.88%). Besides the articles included in this meta-analysis, other notable published studies reporting PCS prevalence data might have been missed due to some limitations in our study, including the suitability of the articles for meta-analysis and the strict inclusion criteria.

The local prevalences reported globally varied, contributing to the high level of possibility for true heterogeneity when meta-analysed. Among the factors causing the variation of the reported prevalence data was the differences of post-COVID-19 assessment timepoints used in each individual studies. Generally, most related published studies reported the prevalence of persisting COVID-19 symptoms at 3, 6, 9, 12, 18 and even 24 months after the onset of acute COVID-19 infection. In this meta-analysis, the follow-up or assessment timepoints were categorized into ≥ 3rd, ≥ 6th, and ≥ 12th months after the index date, whereby the pooled prevalence estimates were 45.06%, 41.30% and 41.32% each, respectively. A cross-sectional study in Malaysia reported that 21.1% or approximately 1 in 5 COVID-19 survivors reported persistent ill health > 3 months post-COVID infection [70]. A study in India reported that 9.4% of people had long-term symptoms after COVID-19 [71]. Two studies in Saudi Arabia by Jabali et al. and Alkwai et al. reported approximately 49% and 51.2% overall PCS prevalence, respectively, while two studies in Turkey by Baris et al. and Kayaaslan et al. reported approximately 27.1% and 47.5% prevalence, respectively [6, 72–74]. In the Republic of South Korea, Kim et al. reported 52.7% prevalence for post-acute COVID-19 syndrome 12 months after COVID-19 infection [75]. A study in Japan reported 56.14% prevalence [76], while a study in Mexico reported high prevalence of 68% at approximately 90 days post-COVID infection [77]. In Canada, Estrada et al. reported 28.5% prevalence of persistent post-COVID-19 symptoms 90 days after infection [78]. A large retrospective cohort study in the UK reported an overall prevalence of 36.55% [8], while another UK study reported that 2.3% of COVID-19 survivors reported symptoms persisting for ≥ 12 weeks [79]. Three different post-COVID studies in Germany reported an overall prevalence of 6.5%, 8.3%, and 49.3%, respectively [80–82]. Boscolo-Rizzo et al. reported that 53% of Italians reported chronic COVID-related symptoms 12 months following the onset of mild to moderate COVID [83], while 59.5% of people in Luxembourg reported at least one symptom 12 months after COVID infection [84]. Two different post-COVID studies in Spain reported 14.34% and 48% prevalence of persistent symptoms at 6 months post-COVID, respectively [85, 86]. In the Netherlands, 12.7% of COVID-19 patients experienced persistent somatic symptoms that could be attributed to COVID-19 after a median 101 days after infection [87]. A cohort study in Switzerland stated that 26% of people with PCR-confirmed SARS-CoV-2 infection reported not having fully recovered after 6–8 months [88]. A prospective cohort study in Russia stated that 47.1% of previously hospitalised patients with COVID-19 reported persistent symptoms at a median 218 days post-discharge [89]. A prospective cohort study in France reported a higher prevalence at 60% [90]. A meta-analysis by Lopez-Leon et al. determined that 80% (95% CI: 65%–92%) of people diagnosed with COVID-19 developed at least one long-term symptom beyond 2 weeks and up to 110 days following acute COVID-19 infection [91]. A review by Chen et al. that meta-analysed post-COVID-19 condition prevalence at 120 days after COVID-19 infection revealed that the estimated global pooled prevalence was 49% (95% CI: 40%–59%) [92]. The review also estimated that the prevalence at 30, 60, 90, and 120 days after COVID-19 infection was 37% (95% CI: 26%–49%), 25% (95% CI: 15%–38%), 32% (95% CI: 14%–57%), and 49% (95% CI: 40%–59%), respectively [92]. Rahmati M. et al. also reported that a total of 41.7% of COVID-19 survivors experienced at least 1 unresolved symptom at 2-year after SARS-CoV-2 infection, and still suffer from either neurological, physical, and psychological sequela [93]. In another meta-analysis by O'Mahoney L. L. et al., which included studies with mean follow-up 126 days post-COVID-19 infection, at least 45% of those survived, went on to experience at least one unresolved symptom, regardless of hospitalisation status [94]. The 41.79% pooled prevalence of PCS worldwide estimated in this review is quite in line with most of the reported pooled-prevalences in other meta-analyses.

### Symptom-specific PCS prevalence

This review mainly focused on determining the pooled prevalence estimate of PCS in general, hence the strict inclusion criteria. In view of the higher bias expectation due to the criteria and keywords set for obtaining the primary outcome of this study, we did not conduct subgroup analyses for symptom-specific pooled prevalence estimates. Compared to the limited number of studies focusing mainly on the overall community-based PCS prevalence, numerous studies have focused on the symptom-specific prevalence estimates related to the conditions occurring post-COVID infection, although the varied terms used based on the initial infection-to-assessment date interval.

Regarding symptom-specific prevalence, the WHO study on the clinical case definition by a Delphi consensus noted that shortness of breath, tiredness, and cognitive impairment are among the typical symptoms of PCS, which might affect daily functioning [95]. A review of the sequelae of other coronavirus infections determined that fatigue, psychological symptoms, and respiratory symptoms were common among SARS and Middle East respiratory syndrome (MERS) survivors [96]. A comprehensive systematic review and meta-analysis reported that the most common symptoms at the 3- to < 6-month assessment were fatigue (32%), shortness of breath (25%), sleep disorder (24%), and difficulty focusing (22%) [97]. Moy et al. stated that the most frequently reported symptoms were fatigue, brain fog, anxiety, insomnia, and depression, with female patients presenting 58% higher probability (95% CI: 1.02, 2.45) of experiencing persistent symptoms [70].

### Sociodemographic-specific PCS prevalence

For sociodemographic-specific prevalence, PCS prevalence was generally higher in the female population. Female patients were less likely to have recovered [88] and were more susceptible to prolonged symptoms compared to male patients [98]. However, some research suggested that there might be a referral bias due to the higher participation in follow-up care by female patients compared to male patients [99]. A cohort study in Moscow reported that women were associated with post-COVID conditions at the 6- and 12-month assessments (OR: 2.04, 95% CI: 1.57–2.65 and OR: 2.04, 95% CI: 1.54–2.69, respectively) [100]. Furthermore, women experienced moderate or severe dyspnoea more often than men (53.8% vs. 21.1%) [101]. Martin-Loeches et al. stated that women were 69% more likely to develop persistent post-COVID-19 symptoms than men [102]. Moreover, most patients with persistent symptoms post-COVID infection were female (63.8%) [22]. In China, women were more likely to experience fatigue and anxiety or depression at the 6-month follow-up after COVID-19 infection [103]. A prospective cohort study in Milan, Italy, reported that women had a threefold higher risk of having persistent COVID-19 symptoms [104]. A few studies suggested that hormones might be involved in perpetuating the hyperinflammatory status of the acute COVID-19 phase in female patients even after recovery [30, 31]. While stronger immunoglobulin G (IgG) antibody production in female patients in the early phase of the illness might contribute to a more favourable outcome therein, it might also be involved in perpetuating disease manifestations [105]. In this study, sex-stratified PCS prevalence was estimated at 47.23% (95% CI: 44.03%-50.42%) in male and 52.77% (95% CI: 49.58%-55.97%) in female, which are in line with the findings from most publications with similar subject.

Populations with comorbidities such as respiratory problems, hypertension, and diabetes also had higher PCS prevalence, which indicated the role of these diseases in influencing the persistence of COVID-19 symptoms. Multiple studies also reported that high body mass index (BMI) was associated with higher hospitalisation rates and increased COVID-19 illness severity, resulting in a higher risk of developing persistent COVID-19 symptoms. Patients with known obese BMI were more likely to experience moderate or severe dyspnoea (37.5%) than those with BMI < 30 kg/m^2^ (27.0%), leading to a higher risk for post-acute COVID-19 [101]. Studies conducted prior to the COVID-19 pandemic era also identified inadequate humoral and cellular immune responses to vaccination against various different viruses in individuals with higher BMI [106, 107]. Another study reported a weak association between obesity and persisting fatigue post-COVID infection [108], even though this might have been due to the higher risk of chronic fatigue among overweight people, particularly obese individuals [109]. Apart from that, hospitalisation during the acute phase might also contribute towards higher PCS prevalence, whereby individuals hospitalised during the acute phase of the infection had higher prolonged symptom prevalence (54%) compared with non-hospitalised patients (34%). In addition to all of the reported cases, there are also a substantial number of undetected infections due to several circumstances, which include silent infections, diagnostic challenges, and underreporting [110–112].

### Geographical region-specific PCS-prevalence

In this review, the estimated pooled prevalence based on continental regions was found highest in Asia (49.79%), followed by America (46.29%), Europe (46.28%), and Australia (42.41%). In a meta-analysis published in April 2022, which had focused on post-COVID-19 condition prevalence at > 28 days after infection, Chen et al. reported that the regional pooled prevalence estimates were highest in Asia 51% (95% CI: 37%-65%), followed by Europe 44% (95% CI: 32%-56%), and USA 31% (95% CI: 21%–43%). The regional differences described in another meta-analysis showed that the pooled prevalence among hospitalised population across continents was significantly higher in Europe 62.7% (95% CI: 56.5%–68.5%) compared to both North America 38.9% (95% CI:24.0%–56.3%) and Asia 40.9% (95% CI: 34.5%–47.7%) [94]. There were less studies on PCS prevalence in Australian and African continental regions published compared to Asian, European, and American regions. The fact that Australia is the only country in the Australian continent might be the cause of the smaller number of related publications from the region. For African region, a study included in this review reported that the prevalence of persistent symptoms 3 months following acute SARS-CoV-2 infection was 50.2% in Liberia [59]. Based on a meta-analysis conducted using long-COVID studies with 4-weeks minimum duration after the COVID-19 acute onset, Müller S. A. et. al. reported that the prevalence of long COVID in African countries varied widely, from 2% in Ghana to 86% in Egypt [113]. The scarcity of published studies on this health condition in African region might be due to varied factors influencing the reporting, including inadequate clinical data and diagnostics, accessibility to healthcare services and lack of awareness [113].

### Strengths and limitations

Numerous post-COVID studies did not use similar term to refer to PCS. In this review, the inclusion criteria used in the study selection process allowed more PCS-specific prevalence data to be captured, contributing as a strength to this study. In addition, further few subgroup analyses conducted in this study allows more additional information on PCS prevalence based on the certain factors studied. Among the limitations in this study is that some of the studies potentially relevant for inclusion might not have been identified during the database search or might have been eliminated during the screening process, due to the different keywords and titles used. This review might have been subjected to language bias too, as only articles in English were included. Other limitation might include the issue of the high between-study heterogeneity in the meta-analysis, which might be a true heterogeneity due to various reasons such as differences in the assessment timepoints, the differences of sociodemographic factors worldwide, plus the smaller number of studies in certain geographical regions, such as studies in Australia as it is the only country in the continental region, and studies in resource-poor countries in Africa and certain parts of Asia.

## Conclusions

This meta-analysis determined that the estimated pooled prevalence for PCS worldwide was 41.79% (95% CI: 39.70%-43.88%). The included studies had a significant moderate heterogeneity (I^2^ = 51%, *p* = 0.03). The estimated prevalence could be used in further related comprehensive studies, including more comprehensive analyses stratifying the prevalence based on symptom-specific risk factors too, which might enable the development of a better healthcare management plan for individuals with PCS. The provision of proper health, social, and economic protections for the higher-risk population is essential, as PCS affects population health and concurrently contributes to the higher economic burden on such patients and countries.

### Supplementary Information

Supplementary Material 1.

## Data Availability

Data relevant to the study were included in the article.

## Abbreviations

BMI: Body mass index
CI: Confidence Interval
COVID-19: Coronavirus disease 2019
I^2^: I-squared
JBI: Joanna Briggs Institute
MERS: Middle East Respiratory Syndrome
MeSH: Medical Subject Headings
NICE: National Institute for Health and Care Excellence
PACS: Post-acute COVID-19 syndrome
PCS: Post-COVID Syndrome
PHEIC: Public Health Emergency of International Concern
PRISMA: Preferred Reporting Items for Systematic Reviews & Meta-Analyses
RCGP: Royal College of General Practitioners
RdRp: RNA-dependent RNA polymerase
SARS-CoV-2: Severe Acute Respiratory Syndrome Coronavirus 2
SIGN: Scottish Intercollegiate Guidelines Network
T2DM: Type 2 Diabetes Mellitus
WHO: World Health Organization
WOS: Web of Science
τ^2^: Tau-squared
